# Congenital nephrotic syndrome: is early aggressive treatment needed? Yes

**DOI:** 10.1007/s00467-020-04578-4

**Published:** 2020-05-06

**Authors:** Tuula Hölttä, Hannu Jalanko

**Affiliations:** Department of Pediatric Nephrology and Transplantation, The New Children’s Hospital, University of Helsinki and Helsinki University Central Hospital, Helsinki, Finland

**Keywords:** Congenital nephrotic syndrome, NPHS1, Albumin, Nephrectomy

## Abstract

Congenital nephrotic syndrome (CNS) was primarily considered one disease entity. Hence, one treatment protocol was proposed in the beginning to all CNS patients. Today, with the help of gene diagnostics, we know that CNS is a heterogeneous group of disorders and therefore, different treatment protocols are needed. The most important gene defects causing CNS are *NPHS1*, *NPHS2*, *WT1*, *LAMB2*, and *PLCE1*. Before active treatment, all infants with CNS died. It was stated already in the mid-1980s that intensive medical therapy followed by kidney transplantation (KTx) should be the choice of treatment for infants with severe CNS. In Finland, early aggressive treatment protocol was adopted from the USA and further developed for treatment of children with the Finnish type of CNS. The aim of this review is to state reasons for “early aggressive treatment” including daily albumin infusions, intensified nutrition, and timely bilateral nephrectomy followed by KTx at the age of 1–2 years.

## Introduction

Congenital nephrotic syndrome (CNS) manifests within the first 3 months of age, and is differentiated from infantile nephrotic syndrome, which appears later during the first 1–2 years of life and mostly has a more favorable prognosis [1]. CNS of the Finnish type (CNF; NPHS1, MIM#256300F) was clinically described by Niilo Hallman in 1956 [2] and Reijo Norio reported its autosomal recessive inheritance 10 years later [3]. CNF is defined as heavy proteinuria, severe hypoproteinemia, edema, and secondary manifestations due to heavy proteinuria detected shortly after birth. Familial and sporadic CNS cases were described in infants without Finnish background already in the 1940s and 1950s, suggesting that primary CNS was not a single entity [4]. In 1998, Marjo Kestilä and coworkers identified mutations in the *NPHS1* gene and named the product of the gene as nephrin [5]. Around 90% of the Finnish CNF patients have two truncating *NPHS1* mutations (Fin-major/Fin-major 60%, Fin-major/Fin-minor 20%, Fin-minor/Fin-minor 10%) [5]. Both mutations lead to a total absence of nephrin molecules in the podocyte slit diaphragm and severe damage of this structure [6]. Worldwide, more than 200 *NPHS1* mutations with variable clinical severity have been identified [7–13].

Other important genetic defects affecting the glomerular filtration barrier are found in *NPHS2* (podocin), *WT1* (Wilms tumor protein 1), *LAMB2* (laminin beta 2), and *PLCE1* (phospholipase C epsilon 1) [14–17]. As is the case with *NPHS1*, these gene defects cause mostly isolated CNS and, less often, syndromic disorders. The magnitude of protein losses and development of kidney failure are, however, variable, and the severity of the disease may vary even between patients having the same mutation. Clinical features of severe CNS are failure to thrive, difficulties in controlling hydration status, and complications, like thrombosis or repeated sepsis.

CNF has been considered a prototype of CNS and its management is still challenging [18–20]. Due to the variable severity of the disease, CNS patients have been treated during the past 2–3 decades according to variable protocols including an early bilateral nephrectomy and kidney transplantation (KTx), unilateral nephrectomy in an attempt to delay dialysis and transplantation, and conservative treatment until development of kidney failure [21]. Due to the rarity of CNS and its genetic and clinical variability, there is not enough evidence to define one treatment strategy for all CNS patients. The aim of this review is to state reasons when and why to use “early aggressive treatment,” including intensified nutrition, albumin infusions, timely bilateral nephrectomy, dialysis, and KTx at the age of 1–2 years.

## Evolution of treatment for severe CNS

The outcome of CNS infants was originally dismal and was related to heavy urinary protein losses. Niilo Huttunen published data on 75 Finnish CNF patients followed over the years 1965–1973 [22]. The children had nutritional support but no one received albumin infusions. Edema and/or abdominal distension was noticed in children before 2 months of age. Over half of the children died before 6 months of age, with mean patient survival of 7.6 months. The oldest child survived 2.2 years. Infection was the most common cause of death (31%), 19% died due to thrombotic complications, and in 43%, the cause of death remained unknown (mostly hemodynamic collapse). None of the children developed uremia before their death.

Based on three CNS children transplanted successfully in Minnesota in the early 1970s, KTx was proposed as the only way to provide a permanent cure and good quality of life for these children [23]. Hoyer et al. proposed further that KTx should be performed before kidney failure to decrease early mortality and growth failure [23]. Active treatment, which included high caloric, high protein, and low sodium diet, was offered in Minnesota to infants since 1971 [4]. The infants also received diuretics and IV albumin to allow sufficient fluid intake for caloric needs and to minimize edema. Despite active treatment, 25% of the patients died before they were transplanted, 85% suffered from bacterial infections, all had growth retardation, and 93% showed developmental delay or neurological abnormalities before KTx. Most of the patients underwent bilateral nephrectomy prior to the transplantation and they became dramatically less irritable, more active, and their appetite improved [4]. Mahan et al. concluded that CNS should no longer be considered lethal and that intensive medical therapy followed by KTx should be the treatment of choice [4].

## Intensified treatment protocol for CNF children

The active Finnish treatment strategy for CNF infants was adopted from Minnesota to prevent early death and complications [1]. A three-stage protocol was regarded feasible in treatment of infants with severe CNS: (1) Management of nephrosis from birth to the age of 6–10 months (weight 7 kg), (2) bilateral nephrectomy and peritoneal dialysis (PD) for 3–6 months, (3) KTx with extra peritoneal engraftment when the weight of 10 kg has been reached (usually 1–1.5 years of age).

Due to high frequency of *NPHS1-*associated CNS in our country, intensified treatment is started for all 0–3-month-old infants with severe hypoalbuminemia, heavy proteinuria (after albumin substitution), and clinical signs of nephrosis. The therapy is individually adjusted based on the clinical response and genetic analysis. Especially in infants with a “mild” genotype (missense mutation in one or two of the *NPHS1* alleles, or other mutated genes) and stable clinical status, weaning off the parenteral therapy should be tried after the first few weeks. This, however, rarely succeeds in our patient material.

So far, 130 CNF children have been treated with this protocol. Two (1.5%) of the infants died during the nephrotic stage, seven on dialysis (5.4%), and seven (5.4%) after KTx. In total, close to 90% of the children have survived and managed quite well with their kidney grafts.

### Management of nephrosis

During the first month, the treatment of infants with CNS strives for controlling edema, preventing and treating complications like infections and thromboses, and providing optimal nutrition to allow best possible growth and development (Table 1) [1, 18]. Diet is energy-rich (130 kcal/kg/day) and protein-rich (3–4 g/kg/day). Albumin infusions (1–4 g/kg/day) combined with intravenous furosemide (1–3 mg/kg/day) are initially divided into three 2-h infusions and after a few weeks given as one nightly infusion or two shorter infusions. The goal of the albumin/furosemide infusions is to minimize edema and hemodynamic problems. The dosing is based on weight and other clinical signs—not on plasma albumin levels, which remain low. In case of increased edema, which is common especially during infections, the nightly albumin/furosemide infusions are divided into 4 daily doses to increase diuresis.

**Table 1:** Intensified treatment protocol for children with severe CNS

Nutrition and fluid management	
- Limit fluid intake by need to provide adequate nutrition	
- Avoid unnecessary IV-fluids and salt supplementation	
- Provide diet with high energy intake (100–150 KCAL/kg/day) and high protein intake (3–4 g/kg/day)	
Albumin (20%) infusions	
- Indicated in treatment of symptomatic hypovolemia and edema	
- Initial dose 1–2 g/kg/day divided in three 2-h infusions	
- After few weeks to stable patients: one nightly albumin infusion or two 2–3-h infusions	
- Furosemide 0.5–2 mg/kg is given together with albumin; consider 2 doses together with a long albumin infusion	
RAAS inhibition trial after neonatal period	
- Indicated in patients with missense *NPHS1* mutations (one or both alleles) or mutations in other than *NPHS1* gene	
- Captopril 0.01–0.5 mg/kg twice daily. Increase the dose gradually	
Nephrectomy	
- Early nephrectomy (in weight approx. 7 kg) is indicated if difficulties in the clinical management of the patient (infections, poor growth and development, thrombotic complications, difficulties in controlling hydration status, cardiac problems)	
- Postpone nephrectomy (till weight > 10 kg) if child grows and develops well and shows no severe complications caused by nephrosis	
- Bilateral nephrectomy is indicated before kidney transplantation if significant nephrosis persists	
- Routine nephrectomies are not necessary to all CNS patients	

In practice, parenteral infusions require central vein catheters, which are usually placed at the age of a few weeks. They carry a risk for thrombotic events, especially since children with CNF show hypercoagulopathy, caused by urinary loss of coagulation factors. Intravenous ATIII (50 units/kg) is given before all invasive procedures and sodium warfarin is used in all infants with INR target level of 2–2.5 [18]. We have observed clearly fewer thrombotic complications since systematic use of anticoagulation. In the study of the European Society for Pediatric Nephrology Dialysis Working Group, the incidence of thrombosis in 71 CNS patients treated between 2010 and 2015 was 13% [20]. However, no statistical difference was found between patients on antithrombotic prophylaxis (67% received heparin and 33% warfarin sodium) and those not on prophylaxis. In our experience, immunoglobulin infusions and antibiotic prophylaxis are not sufficient in prevention of infections and are therefore not used routinely [18, 24].

Reduction of protein excretion by ACE inhibitors (and indomethacin) has been achieved in CNS infants with genetic defects [20, 25]. This “antiproteinuric therapy” is worthwhile trying especially in cases with milder protein excretion and/or a missense mutation (amino acid change) in one or both *NPHS1* alleles, or other nephrosis genes. Among our patients, an infant with Fin-major/R743C genotype responded permanently to ACE inhibitor therapy [26]. On the other hand, patients with truncating mutations have never done that. In CNF patients, even “mild” missense mutations may lead to defective trafficking of encoded protein and its absence on the podocyte surface, which resembles the situation with truncating mutations [27].

### Dialysis

Dialysis may be needed for the treatment of CNS children either after the development of kidney failure or after bilateral nephrectomy.

Continuous PD was introduced to infants already in the 1980s [28, 29]. Infections and poor growth were major problems during the early years [30–32]. Subsequently, the results improved, and it became evident that the most important risk factor for mortality in infants is their non-renal comorbidity [33, 34]. Laakkonen et al. prospectively studied 21 Finnish infants starting PD at mean age 0.59 years [35]. CNF was the primary diagnosis in 71% and bilateral nephrectomy was performed in all of them. Catch-up growth was documented in 57%, and 29% had neurological abnormalities since birth which did not progress during PD [35].

During the past years, the use of hemodialysis (HD) has increased in infants and toddlers with kidney failure. HD is feasible also in nephrectomized CNS infants. In particular, patients who will undergo a living-related donor transplantation and live relatively close to the hospital may be treated for a few weeks with HD prior to KTx. HD is a valid treatment also for infants with problems in the PD therapy (infections and technical problems).

### Kidney transplantation

A KTx program for infants was started in Finland after encouraging a report by Mahan et al. [1]. Holmberg et al., in the mid-1990s, reported excellent 3-year results for 32 CNF patients transplanted after bilateral nephrectomy and treated with PD for at least 3 months [1]. Initially, the results of KTx in children less than 2–5 years of age were less impressive than in older recipients, but patient outcome and graft survival improved clearly over the next decades. Laine et al. in 1994 reported comparable results for KTx in children under 5 years of age and older ones [36]. Five years later, Qvist et al. reported excellent 7-year KTx outcome for children under 5 years of age, of which 84% had CNF as the primary diagnosis [37]. Patient survival was 100% and graft survival was equal for children under and over 2 years of age. Two later studies were in accordance with that of Qvist et al., stating that the age does not affect 10-year graft survival [38, 39]. Today, young children have longer half-life of their kidney graft than older children [38, 39].

In most of the children with severe CNS, it is clear that quality of life is better and risk for complications lower after KTx compared with the nephrotic or uremic/dialysis periods. KTx has been performed on young infants with a weight of < 5 kg and adult-sized grafts have been successfully transplanted into infants weighing 7–10 kg [40]. In our experience, KTx with extra peritoneal engraftment can be safely performed for a 10-kg infant.

Thrombotic events used to be a major problem when transplanting small children. Normalization of the coagulation factor levels before KTx (during the dialysis stage), proper placement of the graft (proximal vessel anastomoses), avoidance of compression and circulatory problems by postponing the closure of fascia for a few days after KTx when necessary, and abundant fluid therapy peri- and post-operatively are essential in avoiding thrombotic events. Using this protocol, we have lost one graft (0.7%) due to thrombosis in CNS children, which is less than that reported in 1–12-year-aged children (around 2%) [40].

Another important issue in infant KTx is the risk for Epstein–Barr virus (EBV) infections and post-transplantation lymphoproliferative disease (PTLD) after KTx. A small child who is seronegative for EBV at the time of KTx has increased risk for PTLD. In our center, PTLD was diagnosed in four school-aged children transplanted as infants due to CNS (4%) as compared with three non-CNS children (2%) transplanted after 2 years of age (total 252 children) [40].

## Long-term outcome

During the nephrotic stage, psychomotor delay caused by muscle weakness is common in children with severe CNS [4]. Typically, the children show dramatic improvement in their developmental skills within 1 year after KTx [4]. Also, the cardiac hypertrophy often seen in the nephrotic stage reverses.

Avoidance of neurological complications is crucial in the management of CNS children. Impairment in intellectual performance and motor skills among KTx children has been mainly associated with neurological comorbidity, hypertensive crises, and seizures during dialysis, as well as lower socio-economic status, but they have been observed also in absence of any comorbidities [41, 42]. Haavisto et al. studied 50 Finnish children 6.9 years after their first KTx performed over the years 1993–2008 at mean age of 3.7 years (44% had *NPHS1* as their primary disease) with respect to cognitive difficulties [43]. Children with KTx scored lower in neuropsychological assessment, both for the verbal and visuospatial domains, compared with the controls. Better cognitive outcome was associated with absence of neurological comorbidity, but also with younger age, shorter disease duration, and better long-term kidney function [43]. Even though CNF children are mostly born with a lower gestational age and birth weight, and are transplanted earlier than children with other diagnoses, they scored higher on all measures than children with other diagnoses. Haavisto et al. concluded that children with KTx later in their life experience longer disease duration, which places them at risk for more neurodevelopmental deficits [43].

Infants receiving living or deceased donor grafts have estimated graft survival of at least 80% at 10 years [38]. Thus, many CNS patients transplanted at the age of 1–2 years need a new graft as young adults. The likelihood, however, is not greater compared with those transplanted 2–5 years later [38]. On the contrary, since children with severe CNS are prone to complications, later KTx might negatively impact their later life.

## Conclusions

CNS is not one disease with a single-treatment protocol. The magnitude of urinary protein losses is important in deciding the treatment strategy. In case of massive proteinuria (100–150 g/L), severe hypoproteinemia may easily lead to edema, thrombotic complications, and sometimes hemodynamic compromise. On the other hand, an infant with proteinuria of 5–20 g/L and gradually developing uremia can manage without aggressive parenteral therapy. Whether to use bilateral, unilateral, or no nephrectomy should be based on severity of CNS and the experience in the treating center. They all might work, but bilateral nephrectomy terminates the harmful proteinuria, and active treatment during nephrosis helps children to grow and develop their motor skills. The quality of life is better after KTx than before it. Treatment results have improved over the last decades; therefore, the decision on timing nephrectomy should be made individually. Nephrectomy may be postponed in patients with severe CNS who do not develop any specific complications during the nephrotic period. Kidney transplantation results in infants are today excellent, and according to the latest studies, infants seem to have the best results, and the outcome of CNS infants does not differ from infants with other diagnoses. Hence, it seems justified to perform early KTx in infants with severe CNS.

### Key summary points

1. Not enough evidence is available to define optimal treatment strategy for CNS due to its rarity and large genetic variation in patient population.
2. Early aggressive treatment for patients with severe CNS includes daily albumin infusions, intensified nutrition, and timely bilateral nephrectomy followed by early KTx.
3. Psychomotor delay is related to muscle weakness and KTx improves developmental skills dramatically.
4. Infants have nowadays the best long-term KTx outcome, and results of *NPHS1* patients do not differ from those of infants with other diagnoses than CNS.
5. Short period of protein deficiency during first year of life, combined with short dialysis and early KTx, seems to be less detrimental for neurological development in children with severe CNS; hence, early KTx should be considered.
6. Studies are needed to compare long-term and neurocognitive outcome between children with severe CNS treated aggressively and conservatively.
